# Correlating Photoluminescence and Structural Properties of Uncapped and GaAs-Capped Epitaxial InGaAs Quantum Dots

**DOI:** 10.1038/s41598-018-25841-7

**Published:** 2018-05-14

**Authors:** Arka B. Dey, Milan K. Sanyal, Ian Farrer, Karthick Perumal, David A. Ritchie, Qianqian Li, Jinsong Wu, Vinayak Dravid

**Affiliations:** 1 Surface Physics & Material Science Division, Saha Institute of Nuclear Physics, 1/AF Bidhannagar, Kolkata, 700064 India; 2 Department of Electronic and Electrical Engineering, University of Sheffield, Mappin Street, Sheffield, S1 3JD United Kingdom; 3 Deutsches Elecktronen-Synchrotron, DESY, Notkestrasse 85, 22607 Hamburg, Germany; 4 Cavendish Laboratory, University of Cambridge, J. J. Thomson Avenue, Cambridge, CB3 0HE United Kingdom; 5 Department of Materials Science and Engineering, Northwestern University, Evanston, IL-60208-3108 USA

## Abstract

The understanding of the correlation between structural and photoluminescence (PL) properties of self-assembled semiconductor quantum dots (QDs), particularly InGaAs QDs grown on (001) GaAs substrates, is crucial for both fundamental research and optoelectronic device applications. So far structural and PL properties have been probed from two different epitaxial layers, namely top-capped and buried layers respectively. Here, we report for the first time both structural and PL measurements from an uncapped layer of InGaAs QDs to correlate directly composition, strain and shape of QDs with the optical properties. Synchrotron X-ray scattering measurements show migration of In atom from the apex of QDs giving systematic reduction of height and enlargement of QDs base in the capping process. The optical transitions show systematic reduction in the energy of ground state and the first excited state transition lines with increase in capping but the energy of the second excited state line remain unchanged. We also found that the excitons are confined at the base region of these elliptically shaped QDs showing an interesting volume-dependent confinement energy scaling of 0.3 instead of 0.67 expected for spherical dots. The presented method will help us tuning the growth of QDs to achieve desired optical properties.

## Introduction

Semiconductor quantum dots (QDs), where charge carriers are confined in all three directions on length scales of a few nanometers, resulting in discrete energy levels, are fascinating size-tunable photonic materials providing narrow line-width emissions^1^. In the last few years, significant attempts have been made to apply these QDs in realizing large-scale quantum processors^2^, large-area display^3^, solar cells beyond Shockley-Queisser limit^4^, and electronic-photonic devices^5,6^. Although QDs can be grown by various physical and chemical techniques ranging from Molecular Beam Epitaxy (MBE) to solution processing^7^, epitaxial QDs of semiconductors, grown in MBE on a single-crystal substrate having a specific crystallographic orientation, provide us with well-defined systems to correlate optical and structural properties. Indium-Gallium-Arsenide (InGaAs) QDs on Gallium-Arsenide (GaAs) (001) substrate, is the best candidate for mid and far infrared photodetectors and as the active material in photovoltaic fields and are the most studied system to understand structure-tunable optical emissions. A substantial amount of research work has been done to correlate emission-wavelength, line-shape and the effect of spin-orbit coupling in quantum yield of the QDs with the composition and strain profiles. The shapes of QDs, apart from size and structure tuning, have also been used as a controlling parameter to tune optical properties. The QDs of various shapes have been grown, like ellipsoidal^8^, lens^9,10^, pyramidal^11^, multi-faceted^11^ and quantum rings^12^. In all these studies^6,13–17^ optical signals were obtained from a buried layer, that reduces non-photonic transitions of exposed surface, and measurements of structural parameters namely composition, strain, size and shape were obtained from the capped top layer, with a key assumption that the buried and surface QD layers are identical in nature.

However, it is becoming increasingly clear that this assumption is not in fact valid as In-Ga intermixing during growth of these two layers on GaAs (001) substrate, the buried-layer just above the buffer layer and the top-layer just below capping layer can be quite different. The process of strain release^18^, segregation, faceting, intermixing, and strain-enhanced diffusion between layers of QDs near buffer and capped layers known to vary^19,20^ strongly affecting the confinement length of charge carriers within QDs that determine the characteristics of emitted photons^15–17^. Here we show, for the first time, that both structural and optical properties can be measured from same layer of QDs as a function of capping layer thickness. The atomic force microscopy (AFM) images obtained from uncapped QD sample enabled us to reconfirm the results obtained from synchrotron X-ray scattering measurements.

Grazing incidence diffraction (GID) X-ray measurements^14,21–28^ are ideal non-destructive techniques to extract the structural information of the nanometer-thin layer of QDs averaged over a large area depending on the footprint of the X-ray beam over a sample. GID studies reveal the average values of the structural parameters; composition, interfacial strain, lateral and vertical confinement lengths which are solely responsible for excitonic energy levels within QDs. The availability of intense synchrotron sources delivering nanometer-sized beams would enable us, in the future, to obtain information regarding composition and strain profiles averaged of a few tens of dots or perhaps an individual QD using the methods described in this paper. Here we present the results of uncapped and capped InGaAs QDs samples and the details of data analysis and measurements are given in the method section. It should be mentioned here that the volume of QDs on GaAs substrate is quite small, as a result only GID peaks like the (200), (220), (400) etc can be measured^29^ nondestructively. Measurement of other conventional diffraction peaks require much higher incidence angle and GaAs peaks overshadows the QD data as the X-ray beam penetrates deeper into the substrate. We optimized the sensitivity of QD signal by varying the incident angle and available energy of the X-ray beam for these measurements. The ratio of integrated intensity of the (200) and (400) profiles from GaAs to InAs in-plane reflection are measured here as a function of in-plane lattice parameters to extract indium profile within an average quantum dot.

**Figure 1:**
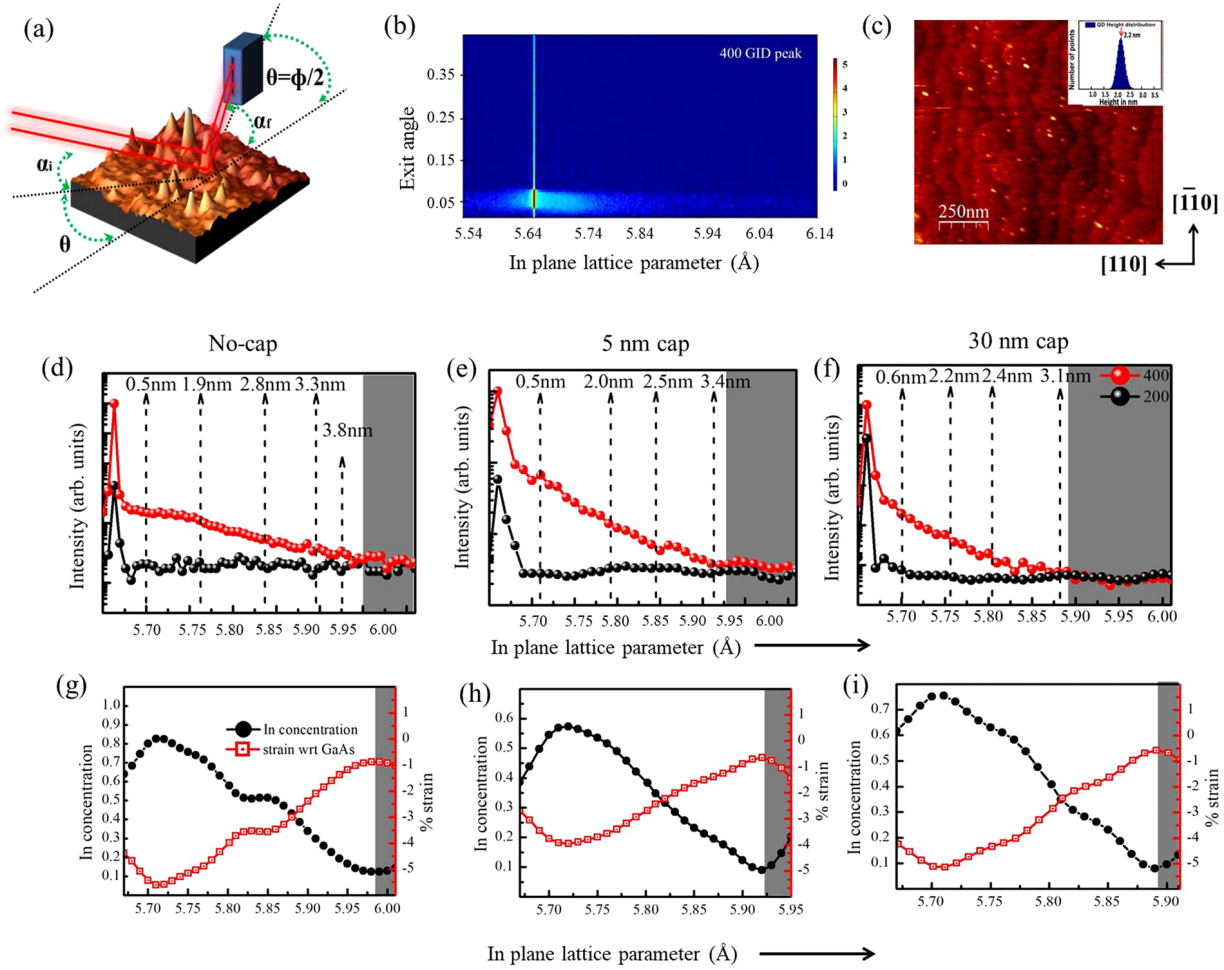
(**a**) Grazing incidence diffraction geometry used in the experiment. Here ${\alpha }_{i}\,$ is kept near the critical angle of the substrate and θ is the incident in-plane angle on (400) and (200) planes and ɸ is the detector angle. Red arrows indicate directions of X-ray beams and the surface of uncapped QDs as seen in AFM is shown as sample. (**b**) GID intensity profile of InGaAs QDs on GaAs substrate around the (400) lattice plane of GaAs in which horizontal and vertical axes denote in-plane lattice parameter and exit angle of the Mythen detector respectively. (**c**) AFM image of uncapped InGaAs QDs shows elongation in [100] direction. Profile of scattered intensity (integrated over exit angle of Mythen detector) around (400) and (200) Bragg peaks of (**d**) uncapped and (**e**) 5 nm and (**f**) 30 nm GaAs capped InGaAs QDs. The in-plane lattice parameters of InGaAs QDs and corresponding heights have been indicated by the dashed line. Indium fraction within InGaAs QDs, in-plane strain with respect to the GaAs for (**g**) uncapped (**h**) 5 nm and (**i**) 30 nm GaAs capped QDs are also shown.

**Figure 2:**
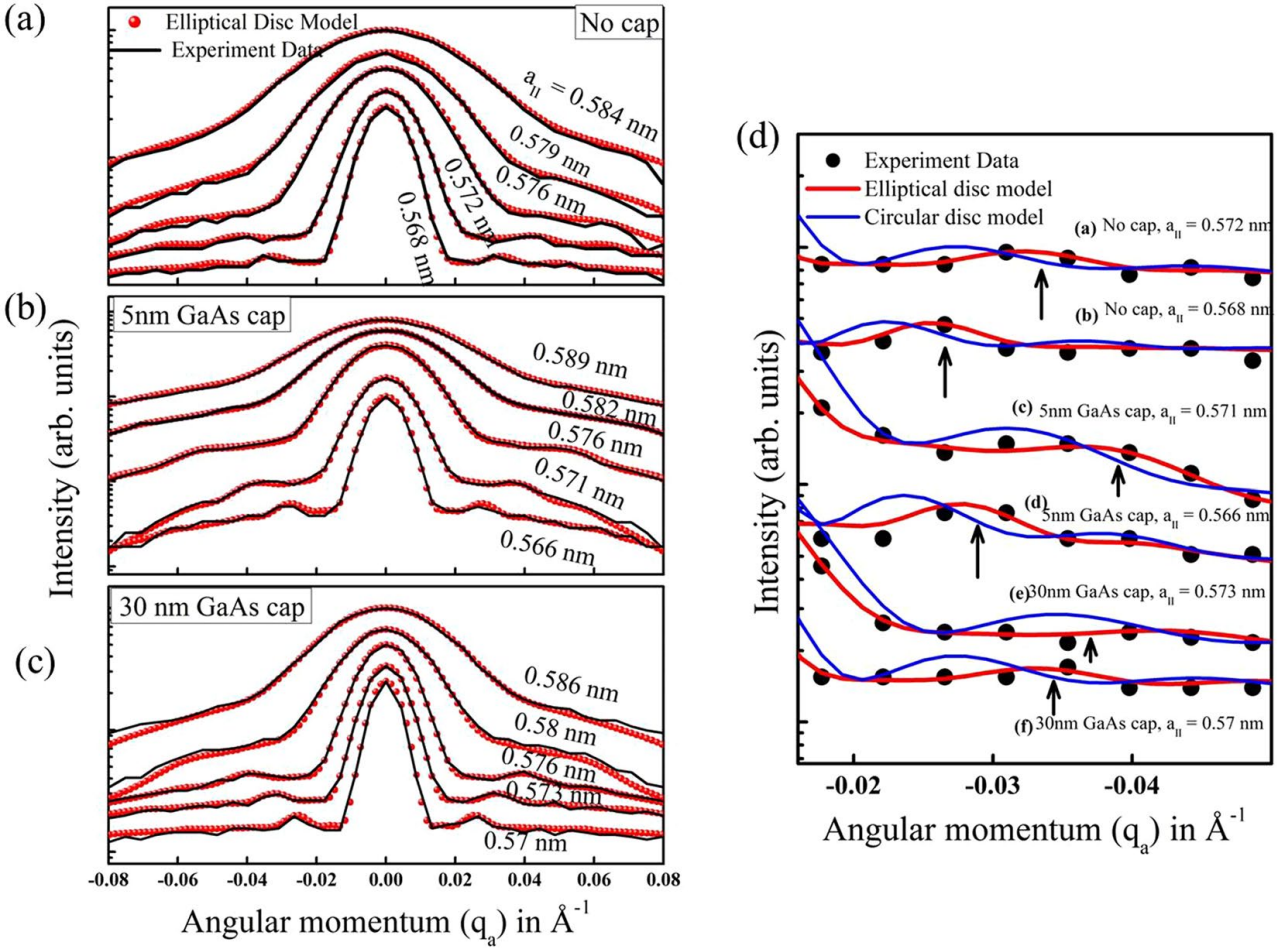
Angular scans (black lines) and fitted profiles (red lines) obtained from elliptical iso-strain disc model for (**a**) uncapped (**b**) 5 nm and (**c**) 30 nm capped QDs. (**d**) Measured angular scan data (black dot) and fitted profiles simulated from elliptical (red line) and circular (blue line) iso-strain disc model for (**a,b**) uncapped InGaAs QDs and (**c**,**d**) 5 nm and (**e**,**f**) 30 nm capped QDs.

The measurements of (400) and (200) in-plane crystal planes of the QDs samples around the GaAs bulk peaks were done by orienting the substrate at an angle ‘*θ*’ with incident X-ray beam and 1D Mythen detector is kept at a detector angle (*Φ*) with respect to direct beam so that it is twice the incident angle of photons to crystal planes (*Φ* = 2*θ*) and the scattering geometry is shown in Fig. 1a. These scans provide direct measure of in-plane lattice parameter (*a*_*II*_) defined in terms of the indices (*h*, *k*, *l*) of the nearest Bragg reflection of the substrate as ${a}_{II}=\lambda \sqrt{{h}^{2}+{k}^{2}+{l}^{2}}/(2\,\sin ({\Phi }/2))\,$. Measured scattering intensity taken by 1D Mythen detector data for different ‘*θ*’ values from the obtained scans have been stitched by *Matlab* programs to generate typical two-dimensional GID images around 400 and 200 Bragg peaks where vertical and horizontal axes denotes exit angle of Mythen detector (*α*_*f*_) and in-plane lattice parameter (*a*_*II*_) respectively. A representative 2D scattered intensity data around (400) Bragg peak of the uncapped InGaAs QDs is shown in Fig. 1b. We could not detect the signature of a pure InAs diffraction peak as the deposition amount of InAs was kept very small (1.8 MLs) and it is known to intermix with GaAs to form InGaAs QDs. Scattered intensity in-between the two extreme *a*_*II*_ values (0.565 *nm* for GaAs and 0.605 *nm* for InAs) exhibit intermixing and alloying of In and Ga within wetting layer (WL) and QDs. The results from AFM measurements presented here clearly show that the InGaAs QDs are ellipsoidal in shape with a preferred elongation in the direction perpendicular to the miscut stair-steps (refer Fig. 1c). We performed both radial and angular momentum scans in GID around in-plane 400 and 200 Bragg diffraction peaks of GaAs, by keeping incidence angle of the X-ray beam below or around the critical angle of GaAs to make the technique sensitive to QDs layer near the surface (refer method section for details). Representative plots of the *I*_400_ and *I*_200_ profiles have been shown for uncapped QDs (Fig. 1d) and capped QDs with 5 nm (Fig. 1e) and 30 nm (Fig. 1f) GaAs. It is to be noted here that in 400 and 200 Bragg peaks contribution of GaAs arises from both the substrate and the cap layer. Scattered intensity reduces towards higher in-plane lattice parameter and reaches background counts when *a*_*II*_ = 0.597 nm for uncapped, *a*_*II*_ = 0.593 nm for 5 nm capped and *a*_*II*_ = 0.589 nm for 30 nm capped QDs. This systematic reduction in data range may be due to X-ray absorption in increasing GaAs cap thickness. The variation of Indium content as a function of in-plane lattice parameter *a*_*II*_ has been calculated using equation (1) given in method section and the obtained profile reveals compositional variation for differently capped QDs. It has been found that the concentration x within an average $I{n}_{x}G{a}_{1-x}As$ QD varies between 0.85 and 0.1 as shown in the plot shown in Fig. 1g for the uncapped sample. For both 5 nm and 30 nm GaAs capped QDs, Indium fraction values reach a maximum of 0.6 and 0.75 respectively as shown in Fig. 1h and i. We shall discuss below the correlation between in-plane lattice parameter *a*_*II*_ and the height of an average QD above the substrate by systematic measurements of Yoneda wings and show that *a*_*II*_ increases toward the tip of the QD as indicated by vertical lines in Fig. 1d,e and f. It is clear from these figures that for all three types of samples, Indium content is higher in the lower and middle region of QDs^19,30^. The in-plane strain profile within QDs with respect to the GaAs were computed using expressions $\,{\varepsilon }_{II}=({a}_{GaAs}-a(x))/a(x)$, as a function of *a*_*II*_ where ${a}_{GaAs}$ and *a*(*x*) are the lattice parameter of GaAs and of InGaAs obtained from Vegard’s law corresponding to Indium content *x*^31^. The calculated strain profiles shown in Fig. 1e and f for QDs with 5 nm and 30 nm GaAs cap layer approach 0% strain at the tip of QDs. The uncapped QDs (Fig. 1g) show in-plane compressive strain of −1% even near the tip region. For all three types of QDs maximum compressive in-plane strain was obtained at the highest Indium containing region, which is about 1 nm height from the substrate. For uncapped, 5 nm and 30 nm capped samples the maximum strain was found to be −5.5%, −4% and −5% respectively (Fig. 1g,h and i). However, out-of-plane lattice parameter and strain can be calculated from the experimentally obtained in-plane lattice parameters by assuming that Poisson’s relation holds here. Fitting of angular scans data for three different types of QDs, namely uncapped and 5 nm and 30 nm capped, samples are shown in Fig. 2a,b and c respectively and fitting parameters are shown in Table 1. The results of the fitting show increase in base dimension of QDs with increasing capping thickness. The dimensions of the base major axes ‘*a*’ for uncapped, 5 nm and 30 nm capped samples came out to be 24 *nm*, 29 *nm* and 32 *nm* respectively. The eccentricity values also increase with increasing capping thickness making buried QDs more elongated (refer Table 1). In Fig. 2d, we have compared the fitting quality of circular and elliptical disc model and it is clear from the figure that elliptical disc model represent the experimental data much better (see equation (3), method section). The circular disc model could not represent the data with any diameter as shown in Fig. 2d.

**Table 1:** Fitting parameters of angular scans: major axes ‘a’ and eccentricity ‘e’ for several ${a}_{II}$.

Surface QDs			5 nm GaAs capped QDs			30 nm GaAs capped QDs		
${a}_{II}$ in nm	a (nm)	e	${a}_{II}$ in nm	a (nm)	e	${a}_{II}$ in nm	a (nm)	e
0.584	8(1), 2.5(0.28)	0.75	0.589	9(1), 2.5(0.31)	0.8	0.586	10(1), 2.7(0.27)	0.8
0.579	10.5(1), 2.7(0.18)	0.77	0.582	11(1), 2.7(0.14)	0.82	0.58	14(1), 2.6(0.17)	0.83
0.576	12.5(1), 2.7(0.09)	0.79	0.576	15(1), 3(0.1)	0.83	0.576	20(1), 3(0.1)	0.86
0.572	18.5(1), 3.3(0.08)	0.81	0.571	21(1), 2.5(0.07)	0.85	0.573	25(1), 3.3(0.8)	0.87
0.568	24, 3(1), 7(0.07)	0.83	0.566	29(1), 2.5(0.05)	0.87	0.57	32(1), 3.5(0.07)	0.89

**Figure 3:**
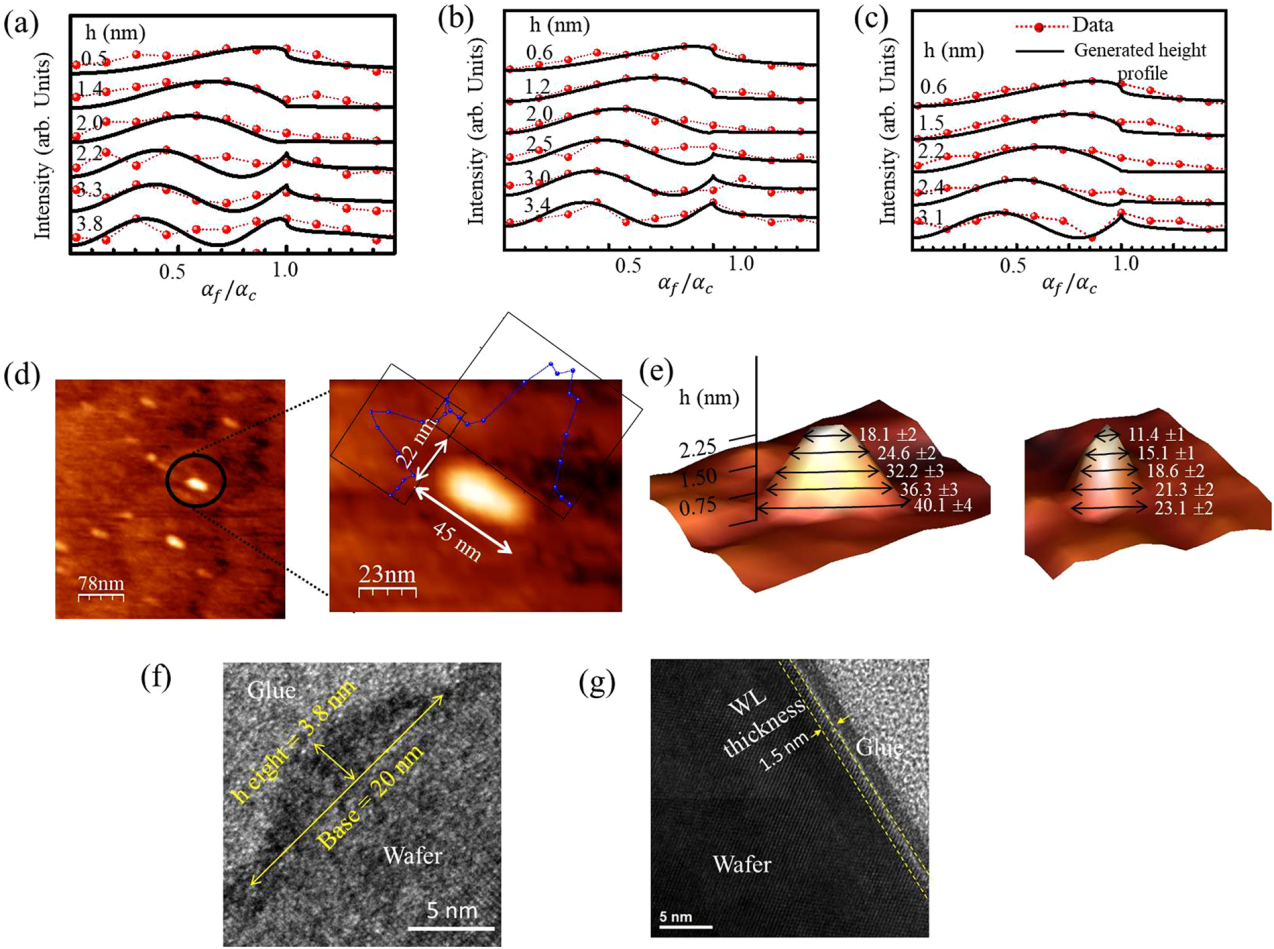
Line profiles of scattered intensity with respect to the ratio of exit angle of Mythen detector and critical angle of substrate for different in-plan lattice parameters of (**a**) uncapped [*a*_*II*_ = 0.57, 0.573, 0.576, 0.58, 0.589, 0.593, 0.596 in nm] (**b**) 5 nm [*a*_*II*_ = 0.57, 0.574, 0.578, 0.584, 0.587, 0.591 in nm] and (**c**) 30 nm [*a*_*II*_ = 0.57, 0.572, 0.576, 0.58, 0.588 in nm] capped QDs [for *a*_*II*_ values top to bottom in the curves]. (**d**) Lateral dimensions of an elongated selected QD from AFM measurements. (**e**) Distribution (over 100 QDs) of lateral dimensions of QDs in two mutually perpendicular directions with heights of uncapped QDs obtained from AFM measurement. (**f**) X-TEM image shows a particular QD having base dimension of 20 nm related to minor axes dimension of QD and height of 3.8 nm, (**g**) X-TEM image shows wetting layer thickness of 1.5 nm indicated by yellow dotted lines.

The results presented in Fig. 3a,b and c for uncapped, 5 nm and 30 nm capped QDs clearly show that QD-base region has in-plane lattice parameter *a*_*II*_ close to GaAs and with increase in average QD height, *a*_*II*_ approach the value of InAs lattice parameter. For uncapped QDs, refer representative data in Fig. 3a, at the heights 0.5 nm, 1.4 nm, 2 nm, 2.2 nm, 3.3 nm, and 3.8 nm above GaAs substrate the *a*_*II*_ values become 0.57 nm, 0.573 nm, 0.576 nm, 0.58 nm, 0.589 nm and 0.593 nm, respectively following equation (5) (refer method section). The data shown in Fig. 3b for 5 nm capped QDs shows a similar trend but the apex region terminates at the *a*_*II*_ value of 0.591 nm at a height of 3.4 nm over GaAs substrate indicating migration of the In-atoms from the apex region of QDs with the growth of GaAs cap-layer. In Fig. 3c the data and fitted profiles for 30 nm cap-layer are shown and the results indicate further reduction in the heights of QDs to 3.1 nm with the maximum measurable in-plane lattice parameter *a*_*II*_ of 0.588 nm.

We have carried out systematic AFM measurement of the uncapped QDs to reconfirm the elliptical shape obtained from the X-ray analysis. A three-dimensional extracted image of an uncapped QD measured in AFM is shown in Fig. 3d – the elliptical base of 44 ± 4 nm and 25 ± 2 nm in the two mutually perpendicular directions can be clearly seen. It is to be noted here that unlike X-ray measurements the AFM can probe only the portions of uncapped QDs outside wetting layer. The average height of QDs was found to be around 2.2 ± 0.4 nm and we shall discuss detailed comparison of the extracted dimensions below to show that the results of AFM measurements are consistent with the results of the analysis of X-ray data. The AFM data from the other two samples could not be collected due to the presence of capping layers having thickness larger than QD heights. In Fig. 3e we have shown cross-sectional views of uncapped QDs obtained from AFM data along the major and minor axes directions at several measured heights of 0.5 nm, 1 nm, 1.5 nm, 1.85 nm and 2.25 nm. At the 0.5 nm height we obtain major and minor axes as 40.1 ± 4 nm and 23.1 ± 2 nm giving the eccentricity of 0.82 ± 0.05. At the tip region with 2.25 nm height these values become 18.1 ± 2 nm and 11.4 ± 1 nm giving lower eccentricity of 0.77 ± 0.04. At the three intermediate heights of 1 nm, 1.5 nm and 1.85 nm, the representative QD have eccentricity of 0.81 ± 0.05, 0.81 ± 0.04 and 0.78 ± 0.04 with decreasing major axes dimensions of 36.3 ± 3 nm, 32.2 ± 3 nm and 24.6 ± 2 nm respectively. Reductions of eccentricity are also clearly observed from base to tip of uncapped QDs in AFM measurements and the obtained values are in reasonable agreement with GID values.

**Figure 4:**
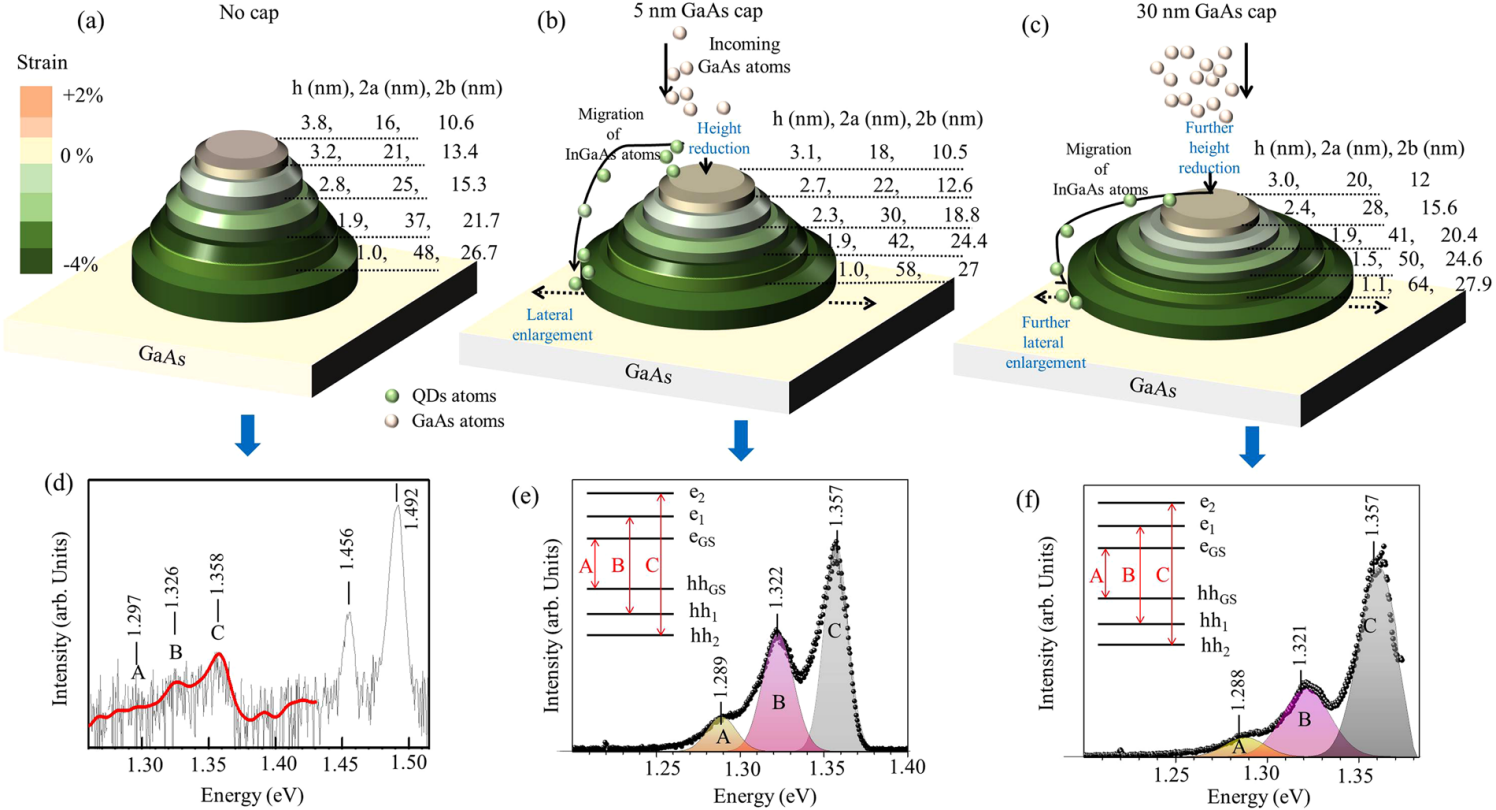
Iso-strain elliptical discs are stacked one over another to form elliptical-lens QD as obtained from radial and angular scans of GID measurements on (**a**) uncapped, (**b**) 5 nm capped, and (**c**) 30 nm capped QDs on GaAs (001) substrate. Color bar indicates the strain values for each elliptical disc. Lateral and vertical dimension of each elliptical disc is indicated as 2a (major axes), 2b (minor axes) and h (height from GaAs substrate). PL response of (**d**) uncapped (red line, smoothed data for guide to eye to improve clarity), (**e**) 5 nm (black line) and (**f**) 30 nm (red line) GaAs capped InGaAs QDs. ‘A’, ‘B’ and ‘C’ are indicates ground and higher excited states excitonic transition lines of QDs and higher energy peaks are related to wetting layer excitonic transition lines.

The dimensions of the major and minor axes at the height of 1 nm in GID data comes out to be 48 nm and 26.7 nm giving eccentricity of 0.83 and only at the height of 1.9 nm we get the values of major and minor axes as 37 nm and 21.7 nm with eccentricity of 0.81, refer results of the uncapped sample shown in Fig. 4a. As mentioned earlier the measured height (Fig. 3e) in AFM was found to be about 1.5 nm less than that obtained from X-ray measurements (Fig. 4a) as AFM cannot access portion of QDs buried under wetting layer. The maximum height the particular QD shown in Fig. 3d and average height (3.8 nm) of uncapped QDs obtained from X-ray data also show this difference. The height of QD obtained from X-ray scattering and AFM measurements can be used to estimate the wetting layer thickness. The QD density is found to be 60 per μm^2^ from AFM data of uncapped quantum dot layer giving average surface area per quantum dot 16667.7 nm^2^. On the other hand calculated base area of an average uncapped quantum dot from X-ray scattering measurements come out to be 1006.6 nm^2^ occupying only 6% of the surface area of entire wetting layer. The composition of $I{n}_{x}G{a}_{1-x}As$ in the base of an average QD (refer discussion on PL below) or that of the wetting layer is around x = 0.25 and 1.8 monolayer of deposited InAs would have given us 2.0 nm of wetting layer if there was no QD formation. An average QD has volume of 3825 (=1006.6 * 3.8) nm^3^ for uncapped QDs layer with an average x = 0.4 and that is equivalent to the volume of 6120 (=3825 * 0.4/0.25) nm^3^ for x = 0.25. This reduces the thickness of the wetting layer of area 16667.7 nm^2^ allocated to each QD to about 1.6 nm - close to the observed difference of QD heights between X-ray and AFM measurements.

We have carried out systematic cross-sectional transmission electron microscopy (X-TEM) measurements^32,33^ to cross-check the dimensions of the QDs and the estimated thickness of the wetting layer. A representative image of an uncapped QD having height of about ~3.8 nm is shown in Fig. 3f. The obtained height values are found to be consistent with the X-ray and AFM results presented here. Base dimension of 20 nm can be related to the minor axes dimension of elliptical-shaped QD. To determine wetting layer thickness we took X-TEM data in between QDs and the thickness of the measured rough surface giving higher contrast was determined to be around 1.5 nm as indicated by yellow dotted lines in Fig. 3g. This value matches well with the estimated wetting layer thickness.

With increasing height of QD, lateral dimensions reduce significantly and major and minor axes dimensions become 16 nm and 10.6 nm with eccentricity of 0.75 at the apex region of QD – this is also consistent with measured AFM value. The strain profile obtained from X-ray measurements has been indicated by the color-bar in the Fig. 4. Similar trends have been observed in the GID data on GaAs capped QDs; obtained results are schematically demonstrated in Fig. 4b and c for 5 nm and 30 nm GaAs capped QDs respectively. Tip region of 5 nm capped QDs obtained from X-ray analysis (refer Fig. 4b) show the average maximum height of QDs to be 3.1 nm with major and minor axes dimensions of 18 nm and 10.5 nm respectively. The shape of the uncapped, 5 nm and 30 nm capped QDs shown in Fig. 3e show much larger lateral dimension as compared to height and these QDs can be approximated as elliptical lenses. We notice increase in lateral size and reduction in height of the QD lenses as the capping thickness increases. For 30 nm capping (refer Fig. 3c) major axis dimension (and eccentricity) becomes 64 nm (0.89) and 20 nm (0.8) at height of 1.1 nm and at the tip region with height 3 nm respectively. The shape changes upon capping have been studied^34,35^ earlier but direct correlations between PL energy and structural parameters were not investigated.

The micro-PL measurements on uncapped and capped $I{n}_{x}G{a}_{1-x}As$ QDs show presence of three transition lines^36^ involving ground state $({E}_{GS}),$ first excited state (*E*_1_) and second excited state (*E*_2_) around 1297 meV, 1326 meV and 1356 meV respectively, as indicated by ‘A’, ‘B’ and ‘C’ in Fig. 4d,e and f. It is known that in-plane compressive strain induced out-of-plane tensile strain breaks heavy-hole (HH) and light-hole (LH) at the Γ point (*k* = 0) degeneracy leading HH-band as effective valence band for optical transition. Therefore ‘A’, ‘B’ and ‘C’ has been correlated with transition lines between ${e}_{GS}-h{h}_{GS}$, ${e}_{1}-h{h}_{1}$ and ${e}_{2}-h{h}_{2}$. Although generally ${E}_{GS}$ peak is expected to be more intense than *E*_1_ and *E*_2_, here we observe stronger *E*_1_ and *E*_2_ lines for all the QD samples. The high incident excitation intensity used here to get micro-PL data may lead to such intensity reversal due to state filling effects^37^. As expected the PL emission from the uncapped QD sample was much weaker due to non-radiative emission process^13^ through surface states, compared to capped samples. We have used 10 points (red line) data smoothening for guide to eye and presented in Fig. 4d both measured and smoothed data -the PL peak positions for uncapped QDs are not affected by smoothing process. The presence of strong heavy-hole free exciton (HHFE) PL emission from InGaAs wetting layer around 1450 meV^38,39^ and similar emission from GaAs around 1490 meV for all the QDs samples were confirmed. The width^38^ of the emissions for all the samples were found to be around 20 meV (refer Table 2) and this value is around half of the width observed in solution processed QDs used for technological applications^2–7^. We observed, except for *E*_2_ emission line, that uncapped sample produces sharper (refer FWHM values Table 2) PL lines.

**Table 2:** Measured PL emission spectrum and results obtained by fitting.

GaAs cap	${E}_{GS}$(meV)	${E}_{1}$(meV)	${E}_{2}$(meV)	${E}_{1}$ – ${E}_{GS}$ (meV)	${E}_{2}$ – ${E}_{1}$ (meV)	FWHM of ${E}_{GS}$ (meV)	FWHM of ${E}_{1}$ (meV)	FWHM of ${E}_{2}$ (meV)	Int. Area of ${E}_{GS}$	Int. Area of ${E}_{1}$	Int. Area of ${E}_{2}$
No cap	1297	1326	1358	29	32	12	14	26	0.28 (6.3%)	1.8 (40.5%)	2.36 (53%)
5 nm cap	1290	1323	1357	33	35	24	26	17	1.46 (10.6%)	5.72 (41.6%)	6.58 (47.8%)
15 nm cap	1289	1322	1357	33	35	19	23	17	0.47 (5.9%)	2.9 (36.3%)	4.6 (57.8%)
30 nm cap	1288	1321	1357	33	36	14	21	16	0.37 (5%)	2.66 (36.4%)	4.37 (58.6%)

**Figure 5:**
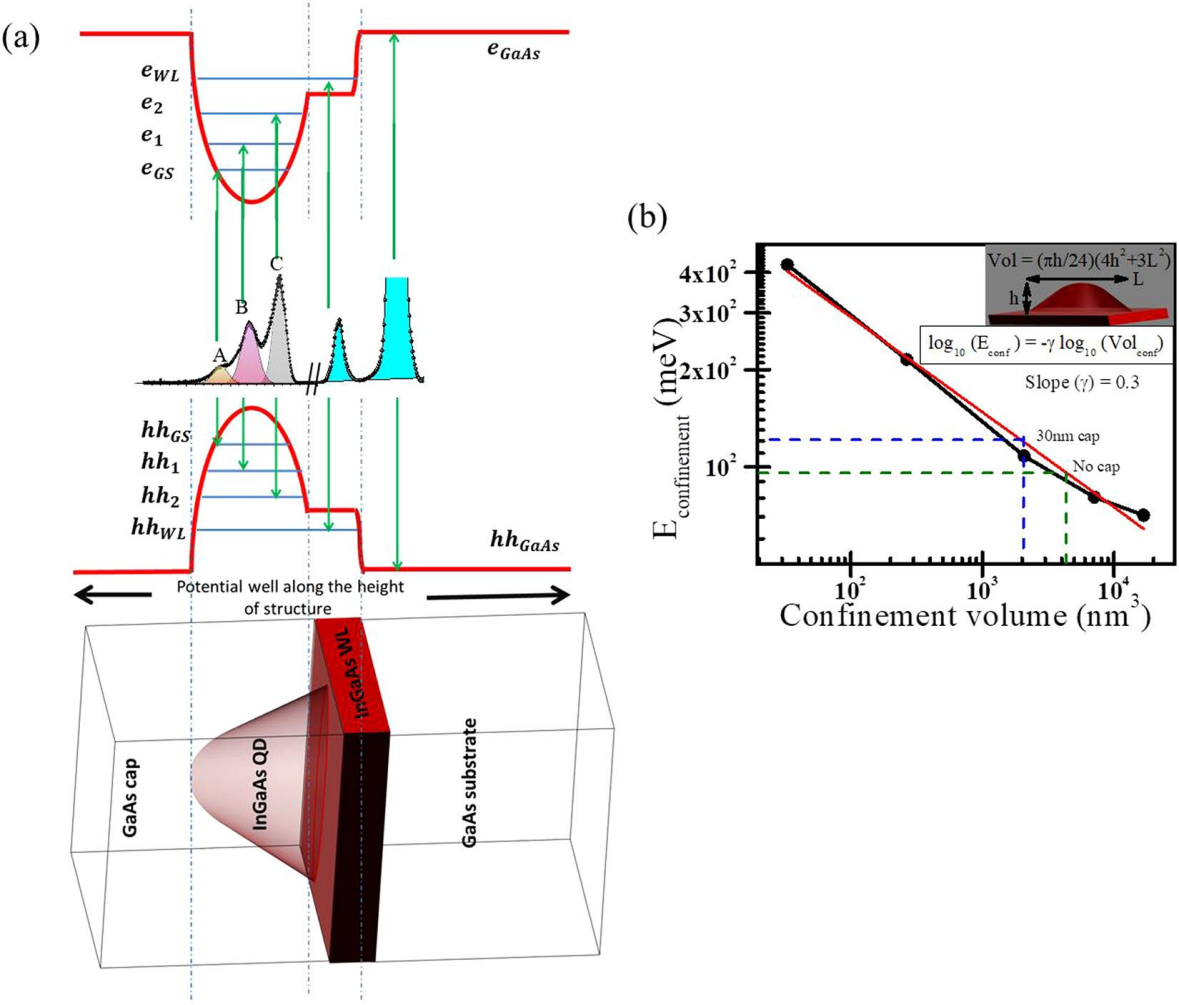
(**a**) Correlation between structure of QDs and obtained PL lines are linked by the excitonic level formation inside the QDs and WLs. Different PL lines (for 5 nm GaAs cap sample) has been associated with the composition and shape dependent exciton levels of QDs. (**b**) The variation of confinement energy with respect to the confined volume for lens shape InGaAs quantum dots. Black dots are the obtained values of electron ground state energy and heavy hole ground state energy^34^. Red line is the least square fitting of the data points which shows slope of 0.3. Blue line and green line indicate the confinement energy values for confinement volume $2049\,n{m}^{3}$ and 4080 $n{m}^{3}$ of lens shaped QDs for 30 nm capped and uncapped sample in GID respectively.

The relationship between QDs structure and confined exciton energy levels are schematically shown in Fig. 5a; the obtained optical transition lines are directly associated with the allowed exciton transitions. The value of *E*_*GS*_ arise from the contribution of (i) composition dependent bulk band gap energy, (ii) confinement size and shape dependent confinement energy and (iii) exciton binding energy. The lateral dimension of the QD elliptical lens becomes larger as the capping thickness increase. The excitonic confinement volume of the average QD elliptical lens can be written as, ${V}_{lens}=\frac{\pi h}{24}(4{h}^{2}+3{L}^{2})$, where L is the lateral confinement dimension and h is the height. It is known that for spherical confinement, ${E}_{confinement}$ is proportional to confinement volume as ${V}_{confinement}^{-\gamma }$ where *γ* is 0.67 due to the fact that ${E}_{confinement} \sim 1/{r}^{2}$ for sphere of radius ‘r’. As the shape of confinement deviate from perfect sphere the power factor (*γ*) reduce from 0.67 to 0.43 (for cuboid shape) and to 0.33 (for pyramidal shape)^34^. ${E}_{confinement}$ for lens shaped QD are calculated by using electron ground state energy and heavy-hole ground state energy^40^; we found *γ* to be 0.3 from the plot of the confinement energy variation with respect to confinement volume (refer Fig. 5b). Confinement energy for lens shaped QDs found to be primarily controlled by the highest lateral confinement dimension, which is the major length of base region of QDs. We obtained the confinement volume of 2049 nm^3^ and 4080 nm^3^ for 30 nm capped and uncapped QDs respectively from GID measurements, which indicate the confinement energy as 121.6 meV and 96.4 meV (refer blue and green lines in Fig. 5b) for 30 nm capped and uncapped QDs respectively. The measured ground state PL emission energy (*E*_*GS*_) can be used to calculate effective composition of $I{n}_{x}G{a}_{1-x}As$ as experienced by exciton within the confined volume *V* of QDs. For 30 nm capped QDs we get ${E}_{GS}=1288$ meV, therefore after subtracting exciton binding energy (~10 meV) and confinement energy, we found composition dependent band gap energy of 1256.4 meV; indicating Indium composition to be *x* = 0.26. Similarly for uncapped QDs, ground state PL energy of 1297 meV gives the value of Indium composition *x* = 0.22. It should be noted here that GID measurements also show larger Indium composition in uncapped QDs as compared to 30 nm capped QDs. The Indium composition obtained from PL measurement was found to be lower than the values obtained in GID measurements. This results indicate that excitons are near the base of the elliptical shaped QDs having lower Indium concentration; the confinement area of the excitons, as discussed below, also matched with the dimension of the base of QDs.

We have carried out systematic Gaussian fittings of all the PL emission lines for all four samples having 0 nm, 5 nm, 15 nm and 30 nm GaAs capping and the resultant parameters are shown in Table 2. For uncapped sample the energy spacing, which are essentially decided by the exciton confinement volume, between *E*_2_, *E*_1_ and *E*_*GS*_ are found to be 32 meV and 29 meV. The energy spacing increases with capping thickness and become 36 meV and 33 meV for 30 nm capped QDs (refer Table 2). The measured separation energy between first excited and ground state energy (*E*_1_ − *E*_*GS*_ = 3*π*^2^*ħ*^2^/2 *mL*^2^) also reconfirmed the consistency of GID and PL measurements. For 33 meV separation observed in 30 nm capped QDs calculated value of L came out to be 60.3 nm - this is consistent with the obtained major axis of the elliptical base of QDs obtained in the GID measurements. The increase in capping thickness shift PL lines towards lower energy as compare to the uncapped sample; for example a shift of 7 meV and 3 meV for *E*_*GS*_ and *E*_1_ was observed for 5 nm capping (Table 2) and for 30 nm capping these shifts were found to be 9 meV and 5 meV respectively (Table 2). However no significant shift could be detected for the *E*_2_ line with capping.

In conclusion, we have reported a direct method to correlate structural and optical properties of epitaxially grown quantum dots with and without capping. The composition profile of an average QD extracted from GID and PL measurements show that excitons are confined near the base of the QDs. We also found that GaAs capping reduces peak concentration of Indium in the QDs. It will be interesting to extend the presented techniques for the solution processes QDs, which are easy to produce and attractive for display application but we need to find a way to orient these QDs in preferred crystallographic directions for such GID measurements. We also plan to use this method to probe smaller numbers of dots in future to reduce the effect of statistical averaging and thereby improve our understanding of structure-property correlation in these emerging materials.

## Methods

### Sample preparation

A set of samples is grown with similar growth conditions but different GaAs capped layer thickness (0–30) nm over InAs QDs on GaAs (001) substrate. The samples were grown by Molecular Beam Epitaxy (MBE). After oxide desorption a 250 nm GaAs buffer layer was grown followed by 100 nm Si-doped GaAs layer to enhance the supply of more photo-excited carriers into the surface of QDs in order to obtain a stronger PL response. The substrate temperature was then lowered to 470 °C during eight minute growth interruption followed by an additional 10 nm of GaAs to separate the QDs from the doped GaAs layer. Then 1.8 monolayers (MLs) of InAs were deposited with a deposition rate of 0.027 µm/h at same temperature with constant As_4_ beam equivalent pressure at $5.8\times {10}^{-6}$ Torr. Different thicknesses of GaAs capping layers were grown over the InAs QDs with growth rate of 0.8 µm/h.

### X-ray GID, AFM, PL and TEM characterization

X-ray measurements were carried out with high beam energy (25 keV) to ensure sufficient X-ray penetration depth to probe buried QDs at Beamline P08 of Petra III, Synchrotron in DESY, Germany. A beam-defining slit of dimension $50\,\mu m\times 300\,\mu m$ was used in vertical and horizontal direction respectively and a position sensitive linear Mythen detector was used to collect scattered intensity. The sample surfaces are imaged by Atomic Force Microscopy (AFM) operating in tapping mode with a nominal tip radius of 10 nm using a Nanoscope-IV multimode SPM. Optical responses of QDs have been examined by micro-PL measurements at low temperature (4 K) with a laser source of wavelength 780 *nm*. The average density of QDs in the X-ray beam foot-print was around 60 per *μm*^2^ as found by AFM measurements. The representative AFM image shown in Figure 1c; exhibit distribution of the epitaxial QDs over the stair-steps of the GaAs substrate. Cross-sectional TEM specimen was prepared by a FEI Helios NanoLab focused ion beam (FIB) system. A field-emission JEOL 2100 F S/TEM equipped with high-angle annular dark-field (HAADF) detector and X-ray energy-dispersive spectrometer (EDS) systems operated at 200 kV, was used for collecting high resolution images.

### Synchrotron X-ray Scattering

The atomic structure factors for 400 and 200 Bragg reflections for zinc blend crystal structure of $I{n}_{x}G{a}_{1-x}As$ alloy present in QDs and WL can be written as ${F}_{400}=x\,{f}_{In}+\,(1-x){f}_{Ga}+\,{f}_{As}$ and ${F}_{200}=x\,{f}_{In}+\,(1-x){f}_{Ga}-\,{f}_{As}$ with ${f}_{In}$, ${f}_{Ga}$ and ${f}_{As}$ as atomic scattering factor for $In$, $Ga$ and *As*. The fractions *x* within QDs and WL as a function of measured *a*_*II*_ values can be calculated by taking ratio of the integrated scattered intensity as,

1 $$\frac{{I}_{400}}{{I}_{200}}={(\frac{x{f}_{In}+(1-x){f}_{Ga}+{f}_{As}}{x{f}_{In}+(1-x){f}_{Ga}-{f}_{As}})}^{2}$$

*I*_400_ and *I*_200_ have been measured by taking integrated scattered intensity over the detector exit angle *α*_*f*_.

Lateral dimensions of QDs have been calculated by several angular scan measurements; each scan has a particular (*θ*, *Φ*) position around the radial intensity profile of (400) GID peak. At each position only incidence in-plane angle ‘*θ*’ was varied by keeping the detector angle fixed. During these scans radial momentum *q*_*r*_ = (4*π*/*λ*) sin (*Φ*/2) remains constant but angular momentum [*q*_*a*_ = (4*π*/*λ*) sin (θ − *Φ*/2)] changes depending on the variation of *θ*. The QDs are generally have been modeled as stacked of iso-strain circular discs^3^ but AFM measurement for the present samples clearly show elongated InGaAs QDs. Our analysis of the radial data presented here clearly shows that stack of iso-strain elliptical discs each having particular in-plane lattice parameters (*a*_*II*_) represent the data much better. The iso-strain length scale has been considered as, *R*(*θ*) = *ab*/(*a* − (*a* − *b*) cos (*θ*)), where ‘*a*’ and ‘*b*’ are the sets of major axes and minor axes respectively. The dimension of the set of (*a*, *b*) values were determined by fitting the measured angular scans data and it was noted that higher values of (*a*, *b*) are associated with base region of QDs and lower values of (*a*, *b*) are linked with apex of QDs. The scattering contribution from each such elliptical disc can be written as,

2 $$I({q}_{a},a,b)=\frac{{I}_{0}}{{\pi }^{2}{a}^{2}{b}^{2}{| < {f}_{InGaAs} > |}^{2}}f({q}_{a},a,b)\,$$

where,

3 $$f({q}_{a},a,b)={[{\int }_{\theta =0}^{2\pi }{\int }_{r=0}^{ab/(a-(a-b)cos\theta )}r{f}_{InGaAs}(r)\exp (-i{q}_{a}r\cos \theta )drd\theta ]}^{2}$$

Here ${f}_{InGaAs}$ is the effective scattering factor. For simplicity we assume ${f}_{InGaAs}$ is independent of *r* and only depend on Indium concentration value *x*. We have assumed^14^ for the fitting of each of *a*_*II*_ radial data that two different discs contribute in the measured intensity profiles. This is known approach^3^ to take care of size distribution of the QDs and the fitted values of two major axes *a* of the fitted discs with relative contribution within bracket are shown in Table 1; for simplicity we have assumed a common eccentricity $e\,=\,\sqrt{1-{b}^{2}/{a}^{2}}$ value for the two discs. It is apparent from Table 1 that contribution of the second discs having very small lateral sizes contribute only 28% to 7% in the intensity and in the subsequent discussion we shall only compare the larger discs obtained from the fitting.

The height from the GaAs substrate of any elliptical discs with fixed *a*_*II*_ can be calculated by analyzing Yoneda wing in the scattered intensity profile as a function of exit angle around 400 Bragg diffraction peak. The height ‘*z*’ above GaAs substrate for a fixed *a*_*II*_ could be calculated from the position of first Yoneda maximum ${\alpha }_{f}^{max\,}$ as

4 $$z=\frac{1}{k{\alpha }_{f}^{max}}{\cos }^{-1}({\alpha }_{f}^{max}/{\alpha }_{c})$$

where *k* denotes the wave number of the X-ray beam and *α*_*c*_ is the critical angle for GaAs substrate. The calculated profiles of the four-process scattering^29–31^ and measured data for some representative *a*_*II*_ for all three QDs samples have been shown in Fig. 3a–c. We have used the expression given below^41,42^, to carry out these calculations.

5 $$I({\alpha }_{m},{z}_{m})=2+2(2{\alpha }_{m}^{2}-1)\cos (2{\alpha }_{m}{z}_{m})+4{\alpha }_{m}\sqrt{1-{{\alpha }_{m}}^{2}}\,\sin (2{\alpha }_{m}{z}_{m})\,{\rm{for}}\,$$

*α*_*m*_ > 1and for *α*_*m*_ > 1

6 $$I({\alpha }_{m},{z}_{m})=1+\frac{2{{\alpha }_{m}}^{2}-1-2{\alpha }_{m}\sqrt{{{\alpha }_{m}}^{2}-1}}{2{{\alpha }_{m}}^{2}-1+2{\alpha }_{m}\sqrt{{{\alpha }_{m}}^{2}-1}}+2\frac{{\alpha }_{m}-\sqrt{{{\alpha }_{m}}^{2}-1}}{{\alpha }_{m}+\sqrt{{{\alpha }_{m}}^{2}-1}}\,\cos (2{\alpha }_{m}{z}_{m})$$

In the above expression ${\alpha }_{m}=\,\alpha /{\alpha }_{c}\,$and $\,{z}_{m}=kz{\alpha }_{c}$.

### Data Availability

The datasets generated during and/or analysed during the current study are available from the corresponding author on reasonable request.

## References

[1] Petroff PM (2011). Semiconductor self-assembled quantum dots: Present status and future trends. Adv. Mater..

[2] Horiuchi N (2015). Silicon photonics: Two-qubit logic gate. Nat. Photonics.

[3] Dai X, Deng Y, Peng X, Jin Y (2017). Quantum-dot light-emitting diodes for large-area displays: Towards the dawn of commercialization. Adv. Mater..

[4] Semonin OE (2011). Peak external photocurrent quantum efficiency exceeding 100% via MEG in a quantum dot solar cell. Science.

[5] Pile D (2016). Microprocessor: electronic-photonic chip. Nat. Photonics.

[6] Horiuchi N (2016). Quantum dots: Photon sorter. Nat. Photonics.

[7] Dai X (2014). Solution-processed, high-performance light-emitting diodes based on quantum dots. Nature.

[8] Blokland JH (2009). Ellipsoidal InAs quantum dots observed by cross-sectional scanning tunneling microscopy. Appl. Phys. Lett..

[9] Walther T, Cullis AG, Norris DJ, Hopkinson M (2001). Nature of the stranski-krastavow transition during epitaxy of InGaAs on GaAs. Phys. Rev. Lett..

[10] Leonard D, Krishnamurthy M, Reaves CM, Denbaars SP, Petroff PM (1993). Direct formation of quantum-sized dots from uniform coherent islands of InGaAs on GaAs surfaces. Appl. Phys. Lett..

[11] Bruls DM (2002). Determination of the shape and indium distribution of low-growth-rate InAs quantum dots by cross-sectional scanning tunneling microscopy. Appl. Phys. Lett..

[12] Kleemans NAJM (2007). Oscillatory persistent currents in self-assembled quantum rings. Phys. Rev. Lett..

[13] Saito H, Nishi K, Sugou S (1998). Influence of GaAs capping on the optical properties of InGaAs/GaAs surface quantum dots with 1.5 µm emission. Appl. Phys. Lett..

[14] Sharma M (2015). Density dependent composition of InAs quantum dots extracted from grazing incidence x-ray diffraction measurements. Sci. Rep..

[15] Lemaitre A, Patriarche G, Glas F (2004). Composition profiling of InAs/GaAs quantum dots. Appl. Phys. Lett..

[16] Cusack MA, Briddon PR, Jaros M (1997). Absorption spectra and optical transitions in InAs/GaAs self-assembled quantum dots. Phys. Rev. B.

[17] Jiang H, Singh J (1997). Strain distribution and electronic spectra of InAs/GaAs self-assembled dots: An eight-band study. Phys. Rev. B.

[18] Alonso-Álvarez D (2011). Strain balanced epitaxial stacks of quantum dots and quantum posts. Adv. Mater..

[19] Joyce PB, Krzyzewski TJ, Bell GR, Joyce BA, Jones TS (1998). Composition of InAs quantum dots on GaAs (001): Direct evidence for (In, Ga) As alloying. Phys. Rev. B.

[20] Costantini G (2006). Interplay between thermodynamics and kinetics in the capping of InAs/GaAs (001) quantum dots. Phys. Rev. Lett..

[21] Takahasi M, Kaizu T, Mizuki J (2006). *In situ* monitoring of internal strain and height of InAs nanoislands grown on GaAs (001). Appl. Phys. Lett..

[22] Schmidbauer M (1998). Ordering of self-assembled Si_1−x_Ge_x_ islands studied by grazing incidence small-angle x-ray scattering and atomic force microscopy. Phys. Rev. B.

[23] Rauscher M (1999). Grazing incidence small angle x-ray scattering from free-standing nanostructures. J. Appl. Phys..

[24] Okuda H, Ochiai S, Ito K, Amemiya Y (2002). Grazing-incidence small-angle scattering measurement of Ge islands capped with Si layer. Appl. Phys. Lett..

[25] Darhuber A (1997). High-resolution x-ray diffraction from multilayered self-assembled Ge dots. Phys. Rev. B.

[26] Wiebach T (2000). Strain and composition in SiGe nanoscale islands studied by x-ray scattering. Phys. Rev. B.

[27] Uragami T (2002). Characterization of strain distribution in quantum dots by x-ray diffraction. J. Cryst. Growth.

[28] Rose D, Pietsch U, Gottschalch V, Rhan H (1995). Investigation of InAs single quantum wells buried in GaAs[001] using grazing incidence x-ray diffraction. J. Phys. D: Appl. Phys..

[29] Schroth P (2012). Investigation of buried quantum dots using grazing incidence X-ray diffraction. Mat. Sci. Eng. B.

[30] Kret S (1999). High resolution electron microscope analysis of lattice distortions and In segregation in highly strained In_0.35_Ga_0.65_As coherent islands grown on GaAs (001). J. Appl. Phys..

[31] Sharma M, Sanyal MK, Satpati B, Seeck OH, Ray SK (2014). Anomalous x-ray scattering study of the growth of inverted quantum hut structures in a Si-Ge superlattice emitting strong photoluminescence. Phys. Rev. B.

[32] Ferdos F (2002). Influence of a thin GaAs cap layer on structural and optical properties of InAs quantumdots. Appl. Phys. Lett..

[33] Lee KH (2005). Effect of thermal annealing in the microstructural and the optical properties of uncapped InAs quantum dots grown on GaAs buffer layers. Solid State Commun..

[34] Garcia JM (1997). Intermixing and shape changes during the formation of InAs self-assembled quantum dots. Appl. Phys. Lett..

[35] Saito H, Nishi K, Sugou S (1999). Shape transition of InAs quantum dots by growth at high temperature. Appl. Phys. Lett..

[36] Leon R (1996). Effects of interdiffusion on the luminescence of InGaAs/GaAs quantum dots. Appl. Phys. Lett..

[37] Raymond S (1996). State filling and time-resolved photoluminescence of excited states in In_x_Ga_1−x_As/GaAs self-assembled quantum dots. Phys. Rev. B.

[38] Patanè A, Polimeni A, Capizzi M, Martelli F (1995). Linewidth analysis of the photoluminescence of In_x_Ga_1−x_As/GaAs quantum wells (x = 0.09, 0.18, 1.0). Phys. Rev. B.

[39] Paskov PP (2000). Photoluminescence up-conversion in InAs/GaAs self-assembled quantum dots. Appl. Phys. Lett..

[40] Ngo CY, Yoon SF, Fan W, Chua SJ (2006). Effects of size and shape on electronic states of quantum dots. Phys. Rev. B..

[41] Helfrich M (2012). Growth and characterization of site-selective quantum dots. Phys. status solidi.

[42] Kegel I (2001). Determination of strain fields and composition of self-organized quantum dots using x-ray diffraction. Phys. Rev. B.

